# Radiochemistry and nuclear chemistry workforce in the United States

**DOI:** 10.1002/acm2.13789

**Published:** 2022-09-21

**Authors:** Sergey Y. Tolmachev, John D. Auxier, Mikael Nilsson, Brian A. Powell, Thomas L. Rucker, Ralf Sudowe

**Affiliations:** ^1^ United States Transuranium and Uranium Registries Washington State University Richland Washington USA; ^2^ Los Alamos National Laboratory Los Alamos New Mexico USA; ^3^ Department of Chemical and Biomolecular Engineering University of California Irvine California USA; ^4^ Department of Environmental Engineering and Earth Sciences and Department of Chemistry Clemson University Anderson South Carolina USA; ^5^ Leidos Oak Ridge Tennessee USA; ^6^ Department of Environmental and Radiological Health Sciences Colorado State University Fort Collins Colorado USA

**Keywords:** nuclear chemistry, radiochemistry, workforce

## 7.1 INTRODUCTION

The disciplines of radiochemistry and nuclear chemistry have direct applications in the fields of national security, nuclear medicine, nuclear power production, and environmental management. Although, often, nuclear and radiochemistry are grouped together and many experts work in both areas, the definition for each field is slightly different. For example, radiochemistry may be defined as the application of the phenomena of radioactive decay and techniques common to nuclear physics so as to solve problems in the field of chemistry. In contrast, nuclear chemistry may be defined as the application of procedures and techniques common to chemistry to study the structure of the atomic nucleus. This chapter provides a brief update of the current state of, and critical U.S. needs for, nuclear chemistry and radiochemistry expertise as the *Assuring a Future U.S.‐Based Nuclear and Radiochemistry Expertise* report was published by National Academy of Sciences (NAS) in 2012.^1^

## 7.2 DEFINITIONS OF THE PROFESSION

Traditionally, radiochemists and nuclear chemists have been chemists who hold one or more degrees in chemistry and have taken additional specialized courses and conducted laboratory work in nuclear and radiochemistry. However, more recently, scientists with degrees in closely related fields, such as environmental engineering, nuclear engineering, chemical engineering, nuclear physics, and health physics, have also been considered part of this profession provided that they have taken courses in radiochemistry and performed work with unsealed radioactive material. A key distinction is that an experienced radiochemist is one who has theoretical training in radiation safety and radiochemistry and understands the proper radiation safety protocols needed to handle unsealed radioactive sources and carrier‐free radioactivity. There has been a related trend in how employers are organized: Traditionally, radiochemists and nuclear chemists were assigned to a chemistry department or division; today, many self‐identified radiochemists are assigned to engineering, material science, or health science units. However, despite (or possibly because of) the recent trends in degree(s) and organization employers, the professions remain well defined.

## 7.3 GENERAL CHARACTERISTICS OF THE WORKFORCE

At present, the professions of radiochemistry and nuclear chemistry stratify the attained levels of expertise of their members by degree held, work experience, and other factors. This approach appears satisfactory and appropriate in light of the broad and multidisciplinary scope of work and the small sizes of these professions. Professional board certification in these specialties is not available or required by federal or state regulations and the professions do not formally define “qualified radiochemist” and “qualified nuclear chemist.” However, for the purposes of this report, we shall define two broad categories of professionals, “radiochemists,” and “nuclear chemists,” as those professionals who have attained the minimum degree requirements to enter each respective profession. “Experienced” professionals in each category will be defined as those members who have additionally obtained sufficient postgraduate training and experience to competently perform their duties independently; the training and experience required to attain competence varies strongly with each job's duties.

Given that there is no formal accreditation body for radiochemists, there is no surveillance data on the actual number of working radiochemists; however, current graduation trends can estimate the number entering the workforce. The distribution in the age of the nuclear and radiochemistry workforce in the United States can be represented by a double‐peaked curve: The first peak occurred with those professionals who received their training during the 1960s and 1970s but was followed by a significant decrease in the output of PhD students during the 1980s, 1990s, and early 2000s^2^, ^3^; the second, larger peak in the workforce occurred with the cohort who received their training during the mid‐ to late‐2000s. This recent increase was due to recognition of the importance of the radiochemistry workforce by several federal departments, such as the Department of Energy (DOE), Department of Homeland Security (DHS), and the Defense Threat Reduction Agency (DTRA); this led to increased federal funding that provided support for faculty and training graduate students involved in nuclear and radiochemistry. Based on DHS data alone, there have been 55 PhD graduates in nuclear and radiochemistry since 2011, effectively more than doubling the number of PhD graduates in the field over the last few years.

## 7.4 EDUCATION AND TRAINING PATHWAYS

At present, in the United States, the pathways to enter the professions of nuclear and radiochemistry are by earning BS, MS, or PhD degrees in chemistry or a closely related discipline. Some degree programs include coursework of relevance to nuclear and radiochemistry. Most graduates receive supplemental on‐the‐job training.

### 7.4.1 Organizations involved in education

There are currently no organizations that formulate recommendations on curricula for degrees in radiochemistry and nuclear chemistry or that accredit degree programs specifically in radiochemistry and nuclear chemistry. However, there are a number of organizations involved in the education of radiochemists, with the level of support ranging from providing summer internships and undergraduate or graduate student stipends to providing opportunities for faculty hiring and development. The American Chemical Society (ACS), Nuclear Chemistry Summer School, and Nuclear Forensics Summer Schools offer hands‐on summer courses with radiochemistry content. The DOE National Analytical Management Training Program also provides a hands‐on course in alpha spectroscopy, in addition to a monthly webinar series that has been occurring since 2013 (http://www.wipp.energy.gov/namp/en_content‐30‐trainingedu.html). The webinars have covered a range of topics of interest to radiochemists, including actinide chemistry, environmental radiochemistry and bioassay, the nuclear fuel cycle, nuclear forensics, environmental and regulatory radiochemistry, and other topics. Additional web‐based training programs are offered by the Environmental Protection Agency that describes basic nuclear decay and radiochemistry fundamentals; a listing of radiochemical measurement protocols, including a question‐and‐answer section, are also provided (https://www.epa.gov/cwa‐methods/approved‐cwa‐radiochemical‐test‐methods). A number of graduate student fellowships and scholarships also are available to support graduate student education and research at a variety of points along the student's degree process. Some key opportunities include the Nuclear Nonproliferation International Safeguards Graduate Fellowship Program. This fellowship program provides support to doctoral students in the field of international nuclear nonproliferation and safeguards and has an emphasis on both technical expertise and policy understanding.^4^

### 7.4.2 Undergraduate education

Undergraduate education opportunities in nuclear and radiochemistry are limited across the United States. Although there appears to be no dedicated nuclear or radiochemistry degree programs, a small number of programs offer a BS in chemistry with a concentration in radiochemistry. These programs differ from one another in the number of courses they require for the concentration, ranging from 2 to 9 courses, including both classroom and laboratory components (e.g., Florida Memorial University). In addition, there are many undergraduate programs that offer one or more stand‐alone courses in radiochemistry or closely related areas (e.g., nuclear chemistry, health physics, and actinide chemistry). Many such courses are co‐taught to graduate and undergraduate students, although some are offered solely to undergraduate students.

As only a few universities provide education in the form of dedicated lecture courses or experimental laboratories in nuclear or radiochemistry to undergraduate students, the federal government and professional societies have developed specialized summer schools to fulfill this educational need, for example, the ACS/DOE Nuclear Chemistry Summer School, which offers opportunities to 24 students (see Section 7.4.1). The DHS currently collaborates with national laboratories (Los Alamos National Laboratory, Lawrence Livermore National Laboratory, and Pacific Northwest National Laboratory) and universities (Washington State University; University of Nevada, Las Vegas; UMC; and University of Utah) to support the Nuclear Forensics Summer School. This effort provides support to ∼12 students per year, with the program focusing primarily on applied radiochemistry as it applies to nuclear forensics analysis. Finally, the U.S. DOE Nuclear Energy University Partnerships (NEUP) program supports Oregon State University's Undergraduate Radiochemistry Summer School that supports 12 students for 6 weeks. This program focuses more rigorously on training students on the nuclear fuel cycle and reactor chemistry as a subset of radiochemistry.

### 7.4.3 Graduate education

Compared to undergraduate opportunities, the number of educational programs where graduate students can receive training in the area of nuclear and radiochemistry is relatively large. A list of programs is maintained by the ACS Division of Nuclear Chemistry and Technology (NUCL) (http://www.nucl‐acs.org/?page_id=73), which indicates that 26 schools offer some level of education across the entire spectrum of nuclear and radiochemistry. Unlike the undergraduate concentrations in radiochemistry that are taught only within chemistry departments, the graduate students and supervisors who identify their work as nuclear and radiochemistry can be found in a variety of departments. For example, programs such as Clemson University and U.C. Irvine have faculty in their Schools of Engineering that graduate PhD students with expertise in radiochemistry.

Unfortunately, as was pointed out by the National Academies of Science,^1^ the National Science Foundation stopped tracking radiochemistry in 2003 as part of their annual Survey of Earned Doctorates due to the dwindling number of graduates. Nonetheless, as was also mentioned in the NAS report, there now appears to be an upward trend in the number of PhD dissertations that have “nuclear chemistry” listed as the subject area, according to the ProQuest Dissertation and Theses database. Therefore, although the overall number of PhD students who have received rigorous training and graduated in the area of nuclear and radiochemistry has certainly decreased since the 1970s, the current number of students who graduate and have some understanding of nuclear and radiochemistry appears to be relatively stable. Although each program offers a different flavor in education and training, dependent on the specialized area of the faculty advisor, every student with fundamental training in the basics of radiochemistry and some training in handling of radioactive material, with additional training either at an educational institution or on‐the‐job, may qualify as a radiochemist.

In recent years, the NEUP program launched an effort to provide advanced training in radiochemistry to graduate students, with the goal of ensuring that American universities have the best equipment and tools available to educate the next generation of industry leaders. To become part of this effort, a student only needed to be enrolled in a graduate program at the U.S. institution where they were seeking a PhD. Once accepted to the traineeship program, they are able to take a short course in advanced nuclear and radiochemistry, perform an internship at a national laboratory, and return to their home institution to continue to finish their doctoral program. In 2017, $5 000 000 was awarded to students pursuing nuclear degrees and degrees in other programs relevant to nuclear energy under NEUP.^5^

### 7.4.4 Postgraduate training

Opportunities for postgraduate training in radiochemistry are offered by universities, as well as by national laboratories. Although a few universities offer a postmasters track in radiochemistry, the majority of postgraduate training focuses on positions for postdoctoral fellows. Opportunities for postdoctoral positions can be found at U.S. universities and national laboratories, as well as international research centers and universities. Many of the U.S. DOE‐sponsored national laboratories actively recruit senior graduate students for postdoctoral positions in radiochemistry to replenish the ranks of their aging and shrinking workforce. As the national laboratories are also a major employer in the field of radiochemistry, a postdoctoral position is commonly a starting point for graduates to find long‐term employment in the national laboratory complex.

### 7.4.5 Alternate pathways

Alternate pathways by which individuals can enter the professions of nuclear and radiochemistry mainly comprise gaining work experience under the supervision of a qualified nuclear or radiochemist. A prerequisite to the alternative pathway is a BS or higher degree in chemistry or closely related field; largely, this occurs when individuals are employed in the national laboratory complex, enter into government positions where radiochemical expertise is needed, such as working in nuclear fuel fabrication and dissolution, or in nuclear medicine. If they do not seek advanced training, these individuals often obtain the majority of their training whilst on the job (see Section 7.4.6).

### 7.4.6 On‐the‐job training

The majority of radiochemists learn the specifics of their trade through on‐site training and experience, with available positions being filled as a result of cross‐training and transition into the field from other related disciplines (such as nuclear physics, health physics, and physical and inorganic chemistry). Indeed, as the nuclear power industry recruits almost its entire chemistry workforce from BS‐level graduates with chemistry and related degrees and the curricula of BS‐level graduates in chemistry and physics in the United States does not typically emphasize nuclear chemistry or radiochemistry, the industry has come to expect little or no knowledge in radiochemistry or nuclear chemistry from its applicants, and thus recognizes the need to train its own workforce. As a similar pathway exists in the national laboratories, the adequacy of radiochemistry expertise in these programs relies on the effectiveness of knowledge transfer.^1^

### 7.4.7 Professional certification and licensure

At present, licensure and certification for nuclear and radiochemists is neither required nor available. However, some radiochemists may seek certification from the American Board of Health Physicists (ABHP), particularly if they are involved in radiobioassay or dosimetry applications.

### 7.4.8 Continuing education

Continuing education can be obtained after joining the workforce through training courses and web‐based training of specific topics, with much of the knowledge base and critical skills (e.g., methods and applications) in nuclear and radiochemistry being similar across the environmental management and national security areas. Web‐based seminars are being used to deliver training in specific areas. Topics are designed to strengthen the participant in areas of professional practice identified by the nuclear industry or national laboratories, including, but not limited to, actinide chemistry, environmental and bioassay radiochemistry, the nuclear fuel cycle, nuclear forensics, environmental and regulatory issues, radionuclide testing in drinking water, other radionuclide specific radiochemistry, and spectroscopy techniques for radioanalytical chemistry (see Section 7.5 for additional details). Some radioanalytical instrumentation manufacturers also offer training courses to provide levels of expertise in the use of their instrumentation.

## 7.5 PROFESSIONAL ASPECTS OF RELEVANCE TO WORKFORCE SUPPLY

Radiochemistry is, by its nature, a highly interdisciplinary research and work area. Radiochemistry professionals interact frequently with scientists from a variety of different fields, with such interactions occurring through networking in professional organizations, attending conferences and workshops, or informal exchanges. These professional activities play an important role in educating students interested in joining the field, retaining existing professionals, and broadening the relevance of nuclear and radiochemistry.

### 7.5.1 Professional organizations

Many of the individuals who self‐identify as nuclear and radiochemists are members of the ACS NUCL, one of 33 ACS specialty divisions. Other societies within which radiochemists are found include the Health Physics Society (HPS), in particular, within the Decommissioning, Environmental and Radon, and Homeland Security Sections, and the American Nuclear Society (ANS) and its Decommissioning and Environmental Sciences, Fuel Cycle and Waste Management, and Isotopes and Radiation Professional Divisions. Radiochemists have also been involved in the Society of Radiopharmaceutical Sciences. Of all the professional societies, only one, the ABHP, offers certification (see Section 9.6.2).

Professional meetings with relevance to radiochemists include the Radiobioassay and Radiochemical Measurements Conference, which is the continuation of an informal conference series that has held annual meetings for 62 years and provides a scientific forum for professionals to present and discuss their work advancing technologies for detecting, measuring, and analyzing radioactive materials. This conference fosters technical excellence among radiochemists and acts as a platform for the development of common, but essential, technical philosophies and practices that are used by government, academic, and regulatory radioassay laboratories, both nationally and internationally. In addition, the Northern California Section of the ANS sponsors the Methods and Applications of Radioanalytical Chemistry Conference on a triennial basis, which focuses on emerging developments in radioanalytical chemistry and has grown to become a major international forum for the field. Finally, the biennial Conference of the Migration of Actinides and Fission Products in the Geosphere (commonly referred to as Migration) is an international forum to discuss chemical processes controlling the migration behavior of actinides and fission products in natural aquifer systems.

### 7.5.2 Interdependencies with other radiation professions

Radiochemistry is related to the disciplines of analytical chemistry, radiopharmaceutical chemistry, nuclear engineering, and environmental engineering. Radiochemists interact with other radiation professionals through projects and programs related to nuclear medicine and radiotracer applications, homeland security, weapons development, nuclear nonproliferation and arms control, nuclear power, radiation protection and dosimetry, and environmental remediation and management. Other radiation professions involved in these same types of projects include health physicists, nuclear engineers, medical doctors, radiation biologists, and radiation epidemiologists. Radiochemists may also be involved in training radiation professionals in other areas, particularly in the areas of homeland security and nonproliferation detection systems, dosimetry measurements, and environmental monitoring.

## 7.6 CURRENT STATUS AND FUTURE OUTLOOK

Formal entrance into the radiochemistry workforce from the undergraduate level is limited currently to the pipeline from a small number of schools that have an established radiochemist in place and are teaching the course, or from schools that offer radiochemistry to undergraduate students as a by‐product of their graduate programs. However, in conversations with the DOE National Nuclear Security Administration (which sponsors radiochemistry through their NA‐10 and NA‐20 offices), DHS, and DTRA, there has been considerable hesitancy to release the number of students funded on projects related to radiochemistry, at either the undergraduate or graduate level, so that the levels and stability of this supply in workforce entrants are unclear. Unfortunately, one issue in the radiochemistry field is consistent support from stable funding sources. As some radiochemistry programs in the country are robust, loss or a perceived weakness in funding can remove a faculty member's ability to retain a student pipeline; faculty relocation in search of funding also makes it difficult to maintain a constant education pipeline.

## 7.7 SUMMARY AND RECOMMENDATIONS

Radiation chemistry and nuclear chemistry are established and mature professions. Their workforces are small, diverse, multidisciplinary, and vital to meeting our nation's needs in critical areas of, for example, defense, nuclear power generation, nuclear forensics (e.g., verification of compliance to weapons treaties), homeland security (e.g., detection of nuclear contraband), and medical applications (e.g., cardiac scans with radioisotopes). The importance of this workforce is well documented by the National Academies of Science (Council, 2012); however, relatively few hard data are available on the characteristics of the workforce. Although there are indications of a recent increase in the number of postgraduates available to enter the field, it is not evident that this resurgence has compensated for earlier losses in the workforce or that a pipeline of new personnel sufficient to meet the current and future national needs for radiochemistry and nuclear chemistry has been established.

A key recommendation to ensuring a sustainable and adequate radiochemistry workforce is better communication among industrial, governmental, and academic organizations that will define the critical scope of work and identify specific educational and training requirements. Furthermore, effective communication between the end users (industrial and governmental organizations) and educators is necessary in the absence of an overarching accrediting organization. Professional meetings and DOE‐ and DHS‐sponsored web‐based and hands‐on summer training programs can be used to disseminate information and provide further guidance for undergraduate students and early career scientists interested in pursuing a career in radiochemistry or nuclear chemistry. Such programs should help to attract the next generation of radiochemists needed to fill jobs of vital importance to our national interests.

There is a need to identify approaches that will better integrate the efforts of the many professional societies with members who have a nuclear or radiochemistry emphasis or divisions. The NUCL is one of the smaller divisions within the ACS, but several opportunities and activities undertaken within the NUCL are relevant to other organizations, for example, the ANS and HPS. Furthermore, several growth opportunities may exist within alternative industries not typically associated with nuclear sciences. Therefore, identifying tangential paths where nuclear science is being employed could offer a significant area for growth. Some examples include environmental radioactivity, either through cleanup of waste or developing radionuclide tagged compounds to monitor behavior in complex systems. It may be useful to survey the programs for which the Nuclear Regulatory Commission has provided faculty grants and see what alternative research areas are being supported.

The recommendations in the following are consensus expert opinions on actions needed to ensure that the radiation chemistry profession will be able to meet the nation's future needs. The authors intentionally declined to recommend detailed methods, timelines, responsibilities of individual organizations, and funding sources. These complex subjects are outside the scope of this review, and, indeed, the authors were prohibited from activities that could be construed as advocacy.

**The authors recommend the following items to ensure the future adequacy of the nation's professional radiation chemistry workforce**.

1. Educational programs in radiochemistry and nuclear chemistry are small and fragile and need to be stabilized through increasing funding for education and research. This could be accomplished, for example, by fostering new collaborations involving existing radiochemistry professionals working in industry and government laboratories, working with academic faculty and assisting them in aligning their research grant proposals with well‐funded national directives.
2. Efforts should be made to reach out to disciplines outside the traditional chemistry and nuclear engineering, for example, materials science, to begin the broader discussion of the needs and current approaches to exploring materials, chemical behavior, and applied sciences that involve radioactive materials. This might be achieved through leveraging existing conference series and inviting a greater diversity of individuals to participate in panel discussions, for example, relevant academic faculty, national laboratory representatives, industry, government sponsors, and other partners in this field.
3. There is a need for increased surveillance of the professional workforce and its pipelines. This should include establishing a better means of tracking the locations of, and needs for, radiochemistry professionals.
4. The professions, or subspecialties, of radiochemistry and nuclear chemistry should be clearly defined and articulated. Considering that (a) there is no formal certification for a radiochemist and (b) the number of different fields in which radiochemistry is being utilized, it is critical at this point to specifically define the characteristics of an experienced or qualified radiochemist. This will enable educational programs to ensure consistency in the educational and experience requirements needed to work as a radiochemist or nuclear chemist. These will facilitate the collection of accurate surveillance data and to provide evidence of viable career paths to future workers.
5. Increase awareness of lucrative scholarship opportunities, career paths, and jobs of vital importance to our national interests.
6. Outreach efforts should mainly target undergraduate students with the intent of informing students interested in an immediate career in the field or pursuing a graduate degree for additional specialization.
7. Relevant journals should be encouraged to highlight jobs, training, and persons involved in the area of nuclear and radiochemistry, possibly as part of a special edition on the field.

## AUTHOR CONTRIBUTION

All the authors listed have contributed directly to the intellectual content of the manuscript.
